# Ethylene biosynthesis and signal transduction during ripening and softening in non-climacteric fruits: an overview

**DOI:** 10.3389/fpls.2024.1368692

**Published:** 2024-04-26

**Authors:** Meiying Liu, Chaoran Wang, Hongliang Ji, Maoxiang Sun, Tongyu Liu, Jiahao Wang, Hui Cao, Qinggang Zhu

**Affiliations:** ^1^ Key Laboratory of Biochemistry and Molecular Biology in University of Shandong, School of Advanced Agricultural Sciences, Weifang University, Weifang, China; ^2^ College of Enology, Northwest A&F University, Yangling, Shaanxi, China; ^3^ College of Agriculture & Forestry Technology, Weifang Vocational College, Weifang, China; ^4^ College of Horticulture, Northwest A&F University, Yangling, Shaanxi, China

**Keywords:** non-climacteric fruit, ripening, softening, ethylene biosynthesis, ethylene signal transduction

## Abstract

In recent years, the ethylene-mediated ripening and softening of non-climacteric fruits have been widely mentioned. In this paper, recent research into the ethylene-mediated ripening and softening of non-climacteric fruits is summarized, including the involvement of ethylene biosynthesis and signal transduction. In addition, detailed studies on how ethylene interacts with other hormones to regulate the ripening and softening of non-climacteric fruits are also reviewed. These findings reveal that many regulators of ethylene biosynthesis and signal transduction are linked with the ripening and softening of non-climacteric fruits. Meanwhile, the perspectives of future research on the regulation of ethylene in non-climacteric fruit are also proposed. The overview of the progress of ethylene on the ripening and softening of non-climacteric fruit will aid in the identification and characterization of key genes associated with ethylene perception and signal transduction during non-climacteric fruit ripening and softening.

## 1 Introduction

Ethylene (C_2_H_4_), one of the most important phytohormones, is crucial to the entire plant growth and development process, including inducing seed germination, inhibiting stem and root elongation, and promoting fruit ripening, abscission, and senescence (Liu et al., 2015). Ethylene also serves as a key mediator of biotic and abiotic stress responses in plants (Schaller, 2012; Dubois et al., 2018), which has made this phytohormone a research focus in recent years. Numerous studies have demonstrated that ethylene is a key regulator of changes in fruit color, texture, aroma, flavor, and nutritional compounds during ripening (Pech et al., 2012). Based on physiological characteristics such as respiratory rate, ethylene release, and response to exogenous ethylene, fruits can be categorized into two basic types: climacteric and non-climacteric. Climacteric fruits exhibit obvious respiratory peaks before the initial maturation stage, and ethylene release increases accordingly. In contrast, ethylene release during the ripening process of non-climacteric fruits is significantly lower than that of climacteric fruits, and the peak in ethylene release is absent (Chervin et al., 2004; Giovannoni, 2004; Paul et al., 2012; Cherian et al., 2014). Numerous studies concluded that ethylene played a crucial role in regulating the ripening and softening of not only climacteric fruits but also non-climacteric fruits (Chervin et al., 2004; Villarreal et al., 2010; Sun et al., 2013).

Non-climacteric fruits express many ethylene biosynthesis genes as well as a series of ethylene signaling components, such as ethylene receptors (ETRs), the negative regulator constitutive triple response (CTR1), and the transduction factors ethylene insensitive 2 (EIN2) and ethylene insensitive 3 (EIN3), EIN3-like (EIL), and ethylene-responsive factor (ERF) (Osorio et al., 2012). Expression of these important signal transduction elements is upregulated to varying degrees during the ripening and softening processes of non-climacteric fruits, as observed in strawberry (Trainotti et al., 2005; Sun et al., 2013; Qian et al., 2016), grape (Chervin and Deluc, 2010; Muñoz-Robredo et al., 2013; Qian et al., 2016; Ye et al., 2017), orange (Katz et al., 2004; Distefano et al., 2009; Wu et al., 2016; Kashyap and Banu, 2019), loquat (Alós et al., 2017), cherry (Ren et al., 2011; Xanthopoulou et al., 2022), and watermelon (Karakurt and Huber, 2008; Karakurt et al., 2014). These findings indicate that ethylene indeed plays a critical role in regulating the ripening of non-climacteric fruits. In this review, we summarized the existing literature regarding ethylene biosynthesis, ethylene physiology, and ethylene signaling in non-climacteric fruit. This review is aimed at helping researchers understand the regulatory mechanisms underlying the ripening and softening of non-climacteric fruits.

## 2 The multiple members of ethylene biosynthesis in non-climacteric fruits

**Figure 1:**
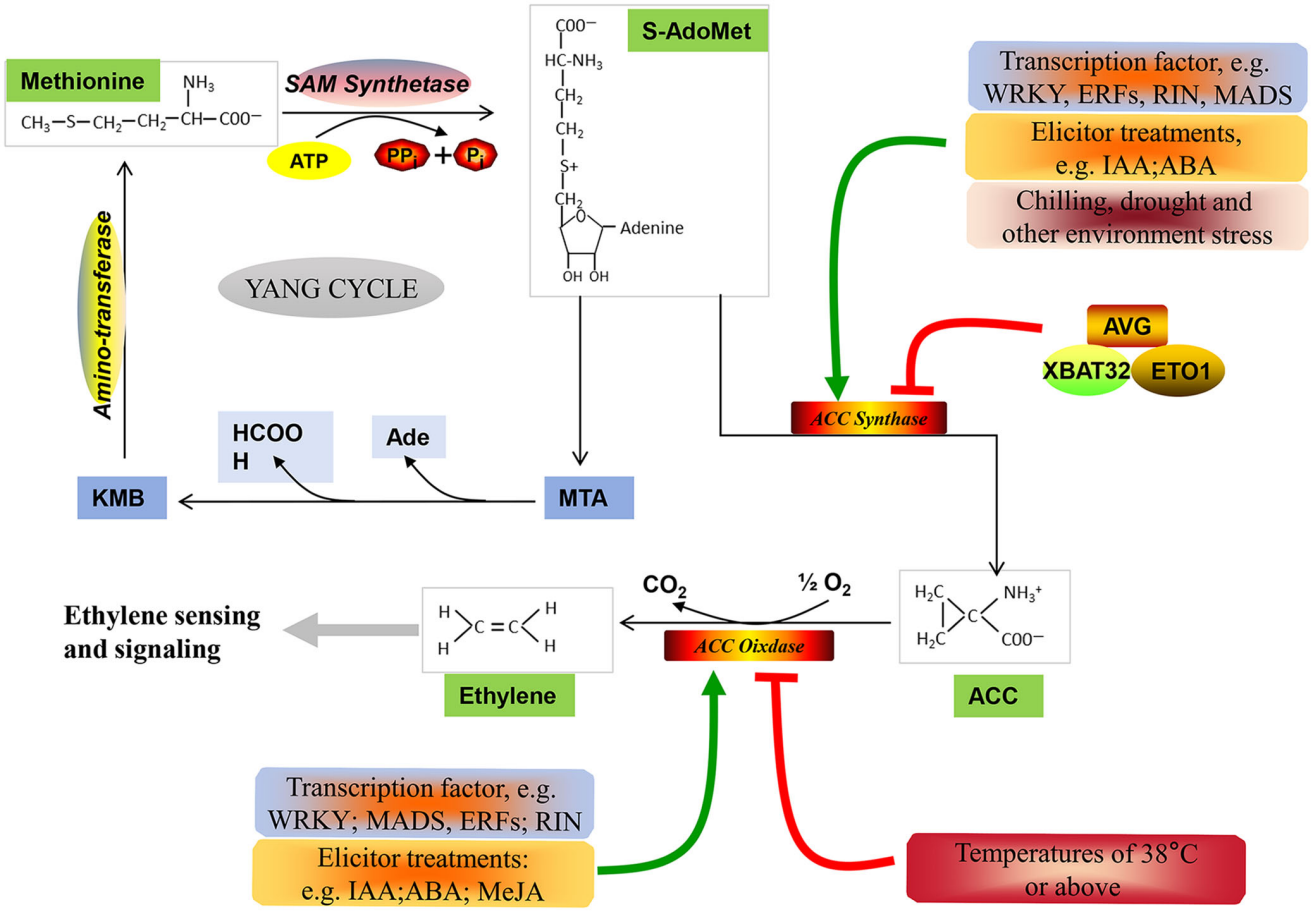
Ethylene biosynthetic pathway. Methionine serves as the precursor of ethylene, and it is catalyzed by SAM synthetase to format *S*-AdoMet (SAM) at the expense of one molecule of ATP per molecule (Bürstenbinder et al., 2007). Subsequently, SAM is metabolized into 1-aminocyclopropane-1-carboxylic acid (ACC) by ACC synthase (ACS) (Pattyn et al., 2021). Additionally, SAM can be diverted to methylthioadenosine (MTA), which can be recycled back to methionine via α-keto-γ-methylthio-butyric acid (KMB) through the Yang cycle (Bürstenbinder et al., 2007). Finally, the catalytic action of ACC oxidase (ACO) converts ACC into ethylene, initiating downstream ethylene sensing and signaling (Yoon and Kieber, 2013). Furthermore, various transcription factors, ubiquitin ligases, regulators, and environmental stresses are involved in regulating the expression of *ACS* and *ACO* expression (Lyzenga and Stone, 2012; Pattyn et al., 2021).

**Figure 2:**
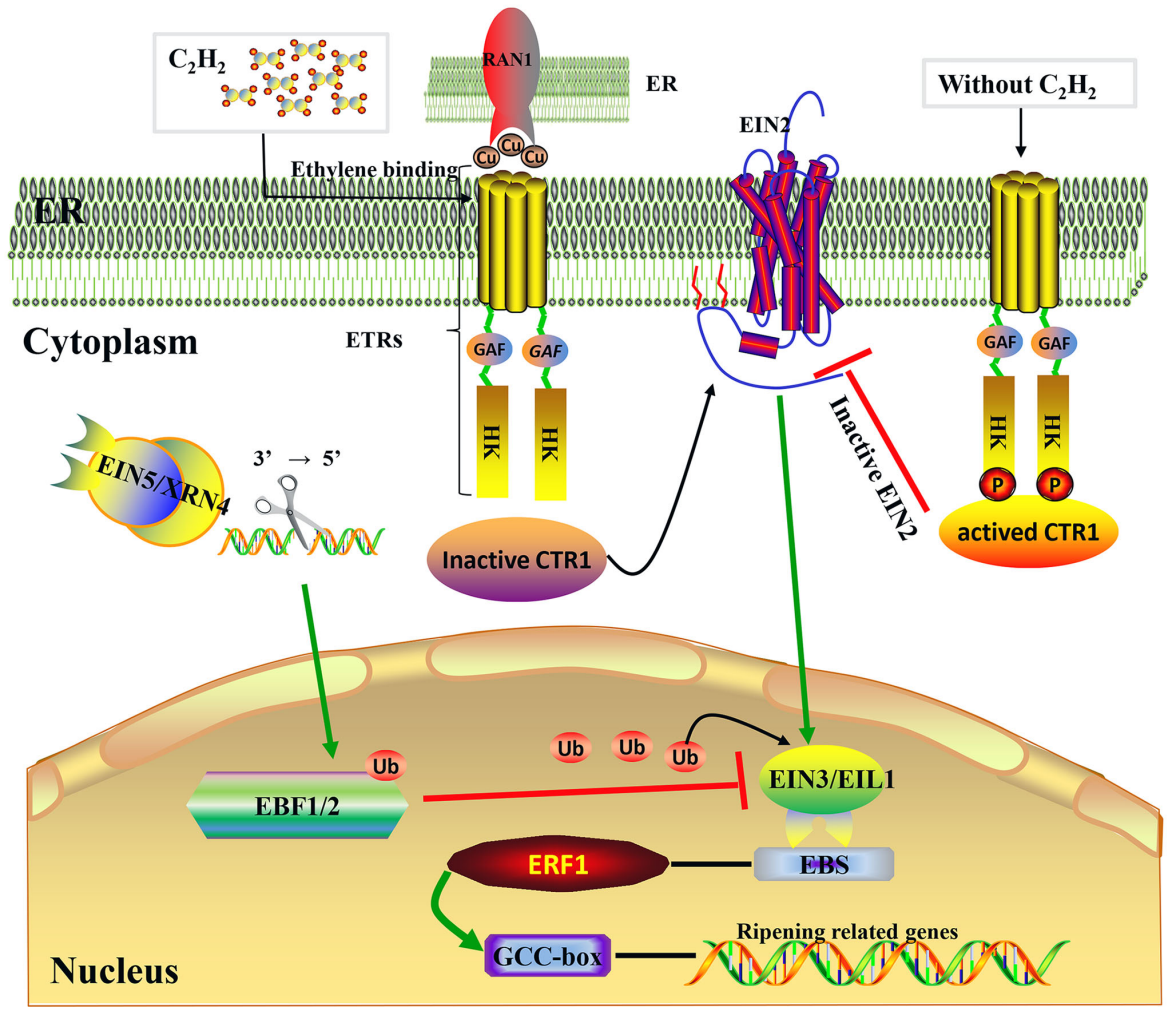
Ethylene signaling transduction pathway. Model of the ethylene signal transduction. Under the mediation of RAN1, which transports copper to ethylene receptors, the presence of ethylene causes the loss of phosphorylation (P) of ethylene receptors (ETRs) at the membrane level (ER) (Binder et al., 2010); following this, the receptor–CTR1 complex is inactivated, the delivery of phosphate groups from CTR1 to EIN2 becomes incapable, and then EIN2 is cleaved and partly enters the nucleus to activate EIN3/EIL1 (Wen et al., 2012). Subsequently, EIN3/EIL1 binds to a conserved motif known as the EIN3 binding site (EBS), which is present within the promoters of ERF1, and this ultimately activates ERF1, which binds to the GCC box in the promoters of many ethylene-inducible, ripening-related genes (Fujimoto et al., 2000). Protein degradation of EIN3/EIL1 is regulated by EBF1/2 via the ubiquitin/26S proteasome pathway, and EIN5/XRN4 5′–3′ exoribonuclease mediated control of EBF1/2 mRNA levels (Potuschak et al., 2003; Olmedo et al., 2006).

Generally, non-climacteric fruits have been classified as a totally separate group from climacteric fruits, characterized by the absence of a typical climacteric ripening pattern. However, comparative genomic studies carried out in climacteric and non-climacteric fruit models suggest that the expression of ethylene biosynthesis- and signaling pathway-related components is common to both climacteric and non-climacteric fruits (Bernales et al., 2019). In this paper, the ethylene biosynthesis pathway (**Figure 1**), the ethylene signal transduction pathway (**Figure 2**), and the functional members of these pathways during the ripening and softening processes of representative non-climacteric fruit models (**Table 1**) are summarized. As shown in **Figure 1**, the involvement of the ethylene biosynthesis pathway during the early ripening period has been confirmed in many reports (Bleecker and Kende, 2000; Glick, 2003; Paul, 2015). Ethylene is produced from the bio-precursor methionine (Met) and is further synthesized into *S*-adenosyl methionine (SAM) via SAM synthetase (Bürstenbinder et al., 2007). Next, SAM is converted into 1-aminocyclopropane-1-carboxylic acid (ACC) by ACC synthase (ACS) via the cleavage of 5′-methylthioadenosine (MTA). ACS, the key rate-limiting enzyme in this pathway, belongs to the pyridoxal-5′-phosphate (PLP)-dependent aminotransferase family and thus requires vitamin B6 as a co-factor (Pattyn et al., 2021), and application of the aminoethoxyvinylglycine (AVG), an inhibitor of ACS activity, could significantly inhibit the evolution of ethylene (Mullins et al., 2010). Afterward, a series of reactions catalyze the conversion of MTA into Met via the Yang or Met cycle (Bürstenbinder et al., 2007). Meanwhile, ACC produces ethylene under the catalysis of ACC oxidase (ACO), thus activating downstream ethylene signaling components and responses (Yoon and Kieber, 2013).

**Table 1:** The functioning members of ethylene biosynthesis and signal transduction in ripening and softening processes for the representative non-climacteric fruit models.

Species	The functioning members	Gene accession no.	Chromosome location	Refs
Grape	VvACS2, VvACS6	GSVIVT00019414001, GSVIVT00018494001	2, 16	Ye et al., 2017
	VvACO1	GSVIVT01015220001	11	Muñoz-Robredo et al., 2013; Ye et al., 2017
	VvETR2	CAN84042	5	Chervin and Deluc, 2010
	VvCTR1-2	GSVIVT01034707001	13	Qian et al., 2016
	*VvEIN2*	GSVIVT01025700001	8	Qian et al., 2016
	VvEIN3-1, VvEIN3-2, VvEIN3-3	GSVIVT01027355001, GSVIVT01037473001, GSVIVT01025324001	13, 6, 6	Qian et al., 2016
	VvEBF1/2	GSVIVT01035856001, GSVIVT01015548001	4, 11	Qian et al., 2016
	VvERF1	GSVIVT01028315001	7	Qian et al., 2016
Strawberry	FaACS1	AY661301	LG6	Merchante et al., 2013
	FaACO2	AJ851829	LG3	Trainotti et al., 2005; Qian et al., 2016
	FaETR2	AJ297513	LG1	Trainotti et al., 2005
	FaCTR1	JQ403286	LG6	Sun et al., 2013
	FaEIN2	gene09749-v1.0-hybrid	LG6	Qian et al., 2016
	FaEIN3-1, FaEIN3-2	gene25474-v1.0-hybrid, gene00379-v1.0-hybrid	LG1, LG7	Qian et al., 2016
	FaEBF1, FaEBF2	gene31045-v1.0-hybrid, gene11633-v1.0-hybrid	LG1, LG1	Qian et al., 2016
	FaERF1, FaERF2	gene11442-v1.0-hybrid, gene08077-v1.0-hybrid	LG4, LG2	Qian et al., 2016
Citrus	*CsACS1*, *CsACS2*, *CsACS3*, *CsACS4*, and *CsACS6*	orange1.1t00416, Cs5g03060, Cs1g21210, Cs3g16400, Cs9g16090	un, 5, 1, 3, 9	Sun et al., 2022
	CsACO1	AJ297350	2	Katz et al., 2004; Wu et al., 2014
	CsETR1, CsERS1	AJ276294, AF092088	3, 3	Katz et al., 2004; Distefano et al., 2009
	CsCTR1	KAH9704377	4	Kashyap and Banu, 2019
	CsEIN2	KAH9735136	6	Kashyap and Banu, 2019
	CsEIL1, CsEIL2	KAH9686827, KAH9790401	5, 2	Kashyap and Banu, 2019
	CsERF1, CsERF4	Cs5g29870, Cs1g07950	3, 7	Wu et al., 2016

Gene accession numbers from this article were cited in the relevant references and can be found in the Genome Database for Rosaceae (strawberry) (http://www.rosaceae.org/), Genoscope (grape) (http://www.genoscope.cns.fr/externe/GenomeBrowser/Vitis/), the Citrus Genome (http://citrus.hzau.edu.cn/), or NCBI GenBank databases.

Throughout the process of fruit development, autocatalytic ethylene production is correlated with increased ACC content as well as increased activities of ACS and ACO. ACS and ACO are encoded by multigene families. In tomatoes, fruit ripening and ethylene production were strongly repressed in transgenic tomato fruits harboring the hpRNAi-ACO construct and an antisense inhibitor of ACS (Alexander and Grierson, 2002; Behboodian et al., 2012). This indicates that both ACS and ACO are important for controlling ethylene production in climacteric fruit. Although ethylene levels are relatively low in non-climacteric fruits during ripening and softening, some physiological and genetic studies have revealed that ethylene still plays an important role in this process (Paul et al., 2012; Zhou et al., 2016; Fenn and Giovannoni, 2021; Ma et al., 2021c). Meanwhile, the function of the *ACS* and *ACO* genes has also been investigated.

*ACS* belongs to a large multigene family, and at least 9, 3, and 18 *ACS* genes have been separately cloned from grapes, strawberries, and oranges, respectively (Merchante et al., 2013; Qian et al., 2016; Sun et al., 2022). As shown in **Table 1**, *VvACS2* and *VvACS6* play important roles in ethylene biosynthesis in grapes (Ye et al., 2017). In strawberries, three members of the *ACS* family all showed high expression levels during the green-fruit stage (Qian et al., 2016). Furthermore, increases in *FaACS1* expression occur alongside accelerated ethylene biosynthesis, indicating that *FaACS1* may be the key *ACS* gene responsible for regulating ethylene biosynthesis in strawberries (Merchante et al., 2013). In sweet oranges, both *CsACS1* and *CsACS2* may regulate ethylene biosynthesis during the early coloring stage, whereas *CsACS3*, *CsACS4*, and *CsACS6* are upregulated during ripening. Each of these genes plays important roles in maintaining stable, low-level ethylene biosynthesis, which is required for ripening (Sun et al., 2022).

Among *ACO* genes, *VvACO1* expression peaks before and after the véraison stage in grapes, while *VvACO2* and *VvACO3* expressions are observed only after the véraison stage (Muñoz-Robredo et al., 2013). Because there is a slight increase in ethylene production around véraison (Xu et al., 2018), *VvACO1* likely plays a critical role in ethylene biosynthesis in grapes (Ye et al., 2017). In strawberries, the expression levels of *FaACO1* and *FaACO3* continue to rise from the green-fruit stage to the white-fruit stage, followed by a gradual reduction to their lowest levels during fruit ripening and coloring. However, *FaACO2* reaches its lowest expression level during the white-fruit stage and then gradually increases during the ripening stage, resulting in substantially increased ethylene biosynthesis just prior to maturation (Trainotti et al., 2005; Qian et al., 2016). In citrus fruits, *CsACO1* appears to be highly expressed during the system II-like stage, in which an autocatalytic burst of ethylene production accompanies the fruit ripening process (Katz et al., 2004; Wu et al., 2014).

Additionally, the expression of *ACO* and *ACS* genes is regulated by multiple transcription factors, including MADS-box, ERF, NAC, and WRKY (Liu and Zhang, 2004; Zhijin et al., 2009; Qin et al., 2012; Xiao et al., 2013; Kou et al., 2016), and the RING E3 ligases, XBAT32 and ETO1, also mediate the proteasomal degradation of ACS proteins in the regulation of ethylene production (Lyzenga and Stone, 2012). Moreover, biotic stressors such as pathogenic bacteria and phytohormones, as well as abiotic stressors such as drought, cold injury, and high temperature, also affect the expression of *ACO* and *ACS* genes (Tsuchisaka and Theologis, 2004). However, research on the transcription factor and ubiquitin ligase-mediated regulation of *ACO* and *ACS* genes in non-climacteric fruit is currently rare, and we suggest that this subject deserves more attention.

## 3 The mediators of the ethylene signal transduction pathway in non-climacteric fruits

In recent years, molecular genetic studies on the model plant *Arabidopsis thaliana* have established the signal transduction pathway underlying the plant response to ethylene. This response occurs when receptors bind ethylene and then send a signal along a linear pathway that comprises MAPK and transcriptional cascades: C_2_H_4_ → ETRs → CTR1→ EIN2→ EIN3/EIL1→ ERF1→ downstream genes. As shown in **Figure 2**, the same transduction mechanism is found in both non-climacteric and climacteric fruits (Bernales et al., 2019).

ETRs are upstream elements and play a negative regulatory role in the entire ethylene signal transduction pathway. In the absence of ethylene, ETRs activate the downstream raf-like serine/threonine kinase CTR1, thus inhibiting the expression of relevant ethylene-inducible genes. However, in the presence of ethylene, ethylene molecules bind to ETRs located at the endoplasmic reticulum membrane, resulting in the dephosphorylation of ETRs. This leads to the inactivation of the ETR–CTR1 complex and the relief of inhibitory activity on ethylene-inducible genes (Tieman et al., 2000). ETR proteins are encoded by various genes, which differ in their structures and expression levels during fruit development, and both mono-deletion and co-deletion of members may induce constituent ethylene responses (Tieman et al., 2000; Cancel and Larsen, 2002; Hall and Bleecker, 2003). At present, three ETR genes (*FaETR1*, *FaERS1*, and *FaETR2*) have been cloned from strawberries, which exhibit differential expression patterns during the fruit ripening process. Specifically, the expression of *FaETR1* and *FaERS1* rises significantly during the ripening stage, while the expression level of *FaETR2* peaks during the white-fruit stage and remains high during the ripening stage. As shown in **Table 1**, *FaETR2* may play an important role in the process of ethylene-mediated fruit ripening (Trainotti et al., 2005). In grapes, at least six ETRs have been identified. Among these, *VvETR1* through *VvETR4* exhibit peak expression before and during the véraison stage, followed by a gradual decline during the ripening process (Chervin and Deluc, 2010; Qian et al., 2016). The transcript abundance of *VvETR2* shows a transient peak at the inception of berry ripening, coinciding with an internal ethylene peak preceding color change (Chervin and Deluc, 2010). By contrast, the transcriptional levels of *VvERS1* and *VvEIN4* gradually rise throughout the late ripening stage until complete maturation (Chervin and Deluc, 2010). Therefore, *VvETR2* may be the key ethylene receptor responsible for grape berry ripening. In oranges, *CsETR1* and *CsERS1* exhibit peak transcriptional levels before and after the degreening stage, indicative of their important roles in fruit degreening and ripening (Katz et al., 2004; Distefano et al., 2009). Additionally, the interaction between ethylene and receptor proteins requires the assistance of copper ions, facilitated by the copper transporter RAN1. Mutants lacking RAN1 cannot bind ethylene due to their inability to transport copper ions (Binder et al., 2010). However, there is limited research on the *RAN1* gene in non-climacteric fruit.

CTR1 is a downstream component of ETRs in the ethylene signaling pathway. *ctr1* mutants exhibit sustained ethylene responses, indicating that CTR1 functions as a negative regulatory element in the ethylene signaling pathway (Huang et al., 2003). In grapes, two CTR1 family genes, *VvCTR1-1* and *VvCTR1-2*, have been identified. *VvCTR1-1* expression remains relatively stable throughout grape berry development, while *VvCTR1-2* expression increases consistently during fruit development and peaks during the véraison stage, followed by a gradual decrease to minimal levels (Qian et al., 2016). Only one *FaCTR1* gene has been identified in strawberries, with peak expression also occurring during the coloring phase (Sun et al., 2013; Qian et al., 2016). Downregulation of *FaCTR1* transcription inhibits the softening and coloring of strawberries, suggesting that *FaCTR1* plays an important role in the process of ethylene-mediated ripening (Sun et al., 2013). In citrus pulp, the *CsCTR1* gene is upregulated throughout development until harvest (Kashyap and Banu, 2019). Furthermore, several lines of evidence indicate that ethylene can modulate the level of ethylene receptor/CTR1 signaling complexes through transcriptional induction (Shakeel et al., 2015). However, there have been limited studies exploring the relationship between ethylene and the level of ethylene receptor/CTR1 signaling complexes in non-climacteric fruit. Additional research is needed to further investigate ethylene-mediated transcriptional and post-transcriptional regulation of CTR1 and ethylene receptor members in these fruits.

EIN2 is located downstream of CTR1 on the endoplasmic reticulum membrane and serves as a positive regulator of the ethylene signaling pathway. *ein2* mutants exhibit total ethylene insensitivity. Moreover, the C-terminal end of EIN2 is thought to participate in signaling output, and ectopic expression of this domain alone can partially activate ethylene responses (Wen et al., 2012). The stability of the EIN2 protein is regulated by two F-box proteins: ETP1 and ETP2 (Qiao et al., 2009). The EIN2 gene, similar to that in *A. thaliana*, has also been cloned in grapes and tomatoes. As shown in **Table 1**, *VvEIN2* expression is minimal during the early developmental period in grapes, rises rapidly during early véraison, and then gradually declines during ripening. In contrast, in strawberries, *FaEIN2* expression is stable throughout the entire developmental process (Qian et al., 2016). In citrus fruits, *CsEIN2* expression remains high until harvest (Kashyap and Banu, 2019). However, further studies should be conducted to evaluate the molecular biological function of the *EIN2* genes in the maturation of non-climacteric fruits.

The ethylene signaling pathway downstream of *EIN2* is mediated by the *EIN3* gene family, including *EIN3* and *EIN3-like 1* (*EIL1*), which act as transcription factors regulating gene expression in cell nuclei. In *A. thaliana*, *ein3* mutants exhibit severely limited ethylene responses, indicating that *EIN3* and *EIL1* play key roles in ethylene signal transduction (Dolgikh et al., 2019). In grapes, three *EIN3/EIL1* gene family members exhibited the same expression pattern during the developmental process. Specifically, their expressions are significantly upregulated 2 weeks before and after the véraison stage and reach the lowest level during the véraison stage (Qian et al., 2016). This is consistent with previous research (Chervin and Deluc, 2010). To date, two *EIN3* members (*FaEIN3-1* and *FaEIN3-2*) have been identified in strawberries, both of which exhibit similar expression patterns to those in grapes. That is, their expression levels are significantly upregulated before and after the coloring stage and decline to their lowest levels during the coloring stage (Qian et al., 2016). These results suggest that the EIN3 family plays an important regulatory role in controlling anthocyanin synthesis and maturation-related changes in non-climacteric fruits. Similar results were reported in sweet orange. As shown in **Table 1**, the transcript abundance of *CsEIL1* and *CsEIL2* increased during fruit enlargement and ripening, suggesting that they may play important roles during the ripening of citrus fruits (Kashyap and Banu, 2019). Through the 26S ubiquitin/proteasome degradation pathway, the EBF1 and EBF2 proteins regulate the stability of the EIN3 and EIL1 proteins (Potuschak et al., 2003). The degradation process takes effect through EIN5/XRN4 with 3′−5′ exonuclease activity, thereby mediating the RNA degradation pathway of these two F-box genes (Olmedo et al., 2006). The expression levels of *FaEBF1* and *FaEBF2* gradually increase during the early development of strawberry fruits but then decline as the fruits ripen. In grapes, the expression of *VvEBF1/2* increases during the expansion stage but significantly decreases during the véraison and ripening stages (Qian et al., 2016). Because EBF1/EBF2 accelerates the degradation of EIN3, the downregulated transcriptional levels of *EBF1/EBF2* during the véraison and ripening stages further suggest that the EIN3 family may potentially play a positive regulatory role in the ripening process of non-climacteric fruits. However, this hypothesis requires further verification.

The ERF family consists of regulatory factors downstream of the ethylene signaling pathway. These factors can be induced by ethylene signaling and either activate or inhibit the transduction and expression of downstream ethylene-responsive genes via binding of their ERF binding domains to GCC-box (AGCCGCC motifs) *cis*-elements (Fujimoto et al., 2000). Numerous studies have indicated that ERF transcription factors are involved in the coloring, ripening, and softening processes of climacteric fruits, including kiwifruit (Zhang et al., 2016), banana (Xiao et al., 2013), pear (Wu et al., 2020; Cheng et al., 2022), and tomato (Liu et al., 2016). However, information about the regulation of ERFs in the development- and ripening-related processes of non-climacteric fruits remains scarce. In grapes, *VvERF1* maintains minimal expression throughout the development process until the late stage of maturation, at which point it is significantly upregulated. However, in strawberries, *FaERF1* and *FaERF2* both exhibit high expression during the early development stage, while their expression gradually declines during the ripening stage (Qian et al., 2016). Similarly, in citrus fruits, genome-wide identification of transcription factors indicates that ERF1 and ERF4 may be important regulators of the late-ripening trait (Wu et al., 2016). Therefore, it is important to investigate the regulatory function of ERF transcription factors, as well as to identify the relevant interacting factors, as they may be linked to ripening and softening in non-climacteric fruits.

## 4 Ethylene-mediated regulation of the ripening and softening of non-climacteric fruits

### 4.1 Ethylene regulates the ripening and softening of strawberries

**Figure 3:**
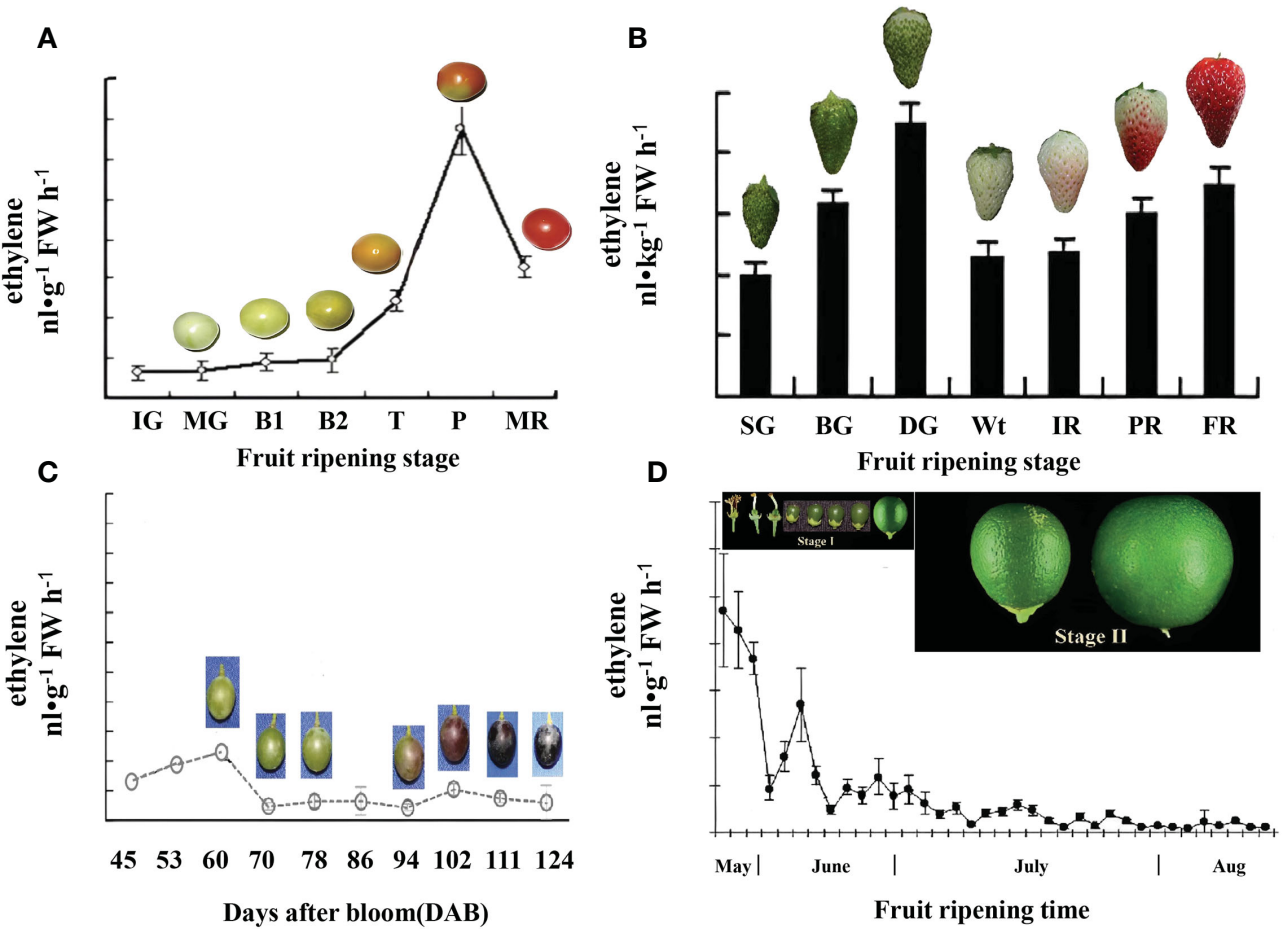
Comparative ethylene evolution in representative climacteric and non-climacteric fruit models. **(A)** Ethylene evolution in the development of the tomato from the immature green to the red ripe stage (Zhang et al., 2009). IG, immature green (20 days after anthesis); MG, mature green (40 days after anthesis); B1, breaker (44 days after anthesis); B2, breaker (45 days after anthesis); T, turning (47 days after anthesis); P, pink (50 days after anthesis); MR, mature red (53 days after anthesis). **(B)** Ethylene evolution in the development of ‘Camarosa’ strawberry fruit during seven stages (Sun et al., 2013), SG (small green), BG (big green), DG (degreening), Wt (white), IR (initially red), PR (partially red), and FR (fully red), which occurred for 7, 15, 20, 23, 27, 31, and 35 days, respectively, after anthesis. **(C)** Ethylene evolution in the development of ‘Moldova’ grape fruit during contentious growth points of berry ripening (Xu et al., 2018). **(D)** Ethylene evolution in the development of attached ‘Valencia’ orange fruit during two growth stages (Katz et al., 2004). Stage I, the cell division stage, starts immediately after fruit set and lasts for approximately 90 days after full bloom (DAFB). Stage II, the cell expansion stage, during which fruit growth continues, mostly by cell expansion, extends until 150–180 DAFB.

In general, climacteric fruits exhibit a dramatic increase in the rate of respiration and ethylene production during the process of ripening. For example, in tomato fruits, ethylene production begins to increase at the turning stage and reaches its peak at the pink stage (**Figure 3A**) (Zhang et al., 2009). By contrast, in non-climacteric fruit, the increase in respiration and ethylene production is limited to a certain extent (Paul et al., 2012). However, recent research has highlighted the important role of ethylene in non-climacteric fruits. For example, in strawberries, although ethylene synthesis is low, the first rapid emission of ethylene occurs in green fruit, and this emission extends to the degreening stage (20 days after anthesis). Additionally, a second slower emission of ethylene occurs following the white stage (23 days after anthesis) (**Figure 3B**), suggesting that ethylene production is likely a key component of the ripening process in strawberries.

Notably, exogenous ethylene affects many important quality attributes in strawberries, including firmness (Jiang et al., 2001; Villarreal et al., 2009, 2016; Elmi et al., 2017), anthocyanin accumulation, phenylalanine ammonia-lyase (PAL) activity (Villarreal et al., 2009; Merchante et al., 2013; Sun et al., 2013), organic acid content (Merchante et al., 2013; Elmi et al., 2017), sugar content (Villarreal et al., 2016; Tosetti et al., 2020), phenolic accumulation (Villarreal et al., 2010; Lopes et al., 2015), and the expression of volatile-related genes (Merchante et al., 2013). In addition, some studies also report that ethephon affects fruit diameter and anthocyanin content, and these effects are dependent on the fruit developmental stage at which the treatment is applied. Ethephon treatment at the green stage of fruits results in a larger diameter, while ethephon treatment at the pink stage results in a smaller diameter. Meanwhile, only treatment of white fruits with ethephon increases the anthocyanin content (Reis et al., 2020). Similarly, another study also reports that ethylene treatment at the green stage of strawberry fruits can delay the accumulation of anthocyanins, as well as downregulate the key anthocyanin biosynthesis genes *FcANS* and *FcUFGT* (Figueroa et al., 2021). Conversely, the use of 1-methyl cyclopropane (1-MCP) at the white stage can significantly inhibit fruit ripening and coloring (Villarreal et al., 2009). Moreover, exogenous ethylene treatment can stimulate the upregulation of the ethylene receptor genes *FaETR1* and *FaERS1*, the ethylene-response factor gene *FaERF2*, and the ethylene biosynthesis gene *FaACO1* (Trainotti et al., 2005; Lopes et al., 2015). Meanwhile, the negative regulatory factor *FaCTR1* also plays an important role in the maturation of strawberry fruits. Constructing an interfering vector to downregulate *FaCTR1* results in the inhibition of strawberry coloring and softening, as well as the facilitation of ethylene biosynthesis (Sun et al., 2013). Thus, it appears that the ethylene signal transduction pathway plays a highly important role in the ripening process of strawberries.

### 4.2 Ethylene regulates the ripening and softening of grapes

Grape is a non-climacteric fruit that does not exhibit a typical respiratory peak and whose maturation apparently does not require ethylene. However, recent research has shown that ethylene plays a critical role in the ripening of grape berries. Grapes exhibit a slight increase in ethylene production around véraison, as shown in **Figure 3C** (Xu et al., 2018). It has been confirmed that the content of endogenous ethylene increases significantly before the véraison stage. During this period, the grape berries expand rapidly, anthocyanin is constantly accumulated, and pulp acidity is reduced (Chervin et al., 2004). Meanwhile, the contents of aroma compounds such as terpinols, ethyl alcohols, and esters increase significantly (Bellincontro et al., 2006).

Exogenous ethylene upregulates the transcription of xyloglucan endotransferase (*XET*) and aquaporin (*AQUA*) genes, thus altering fruit texture and promoting softening (Chervin et al., 2008). Exogenous ethylene also facilitates the early abscission of grape berries (Bessis et al., 2000), promotes anthocyanin accumulation, and induces expression of the anthocyanin synthesis-related genes *CHS*, *F3H*, and *UFGT* (El-Kereamy et al., 2003). Further research has shown that exogenous ethylene induces the expression of anthocyanin regulatory genes, and this is caused by direct action of the ethylene signal transduction pathway on the *UFGT* and *MYBA1* promoters to directly regulate gene expression (Tira-Umphon et al., 2007). Exogenous ethylene also facilitates the production of endogenous ethylene and the expression of *VvACO* genes (Muñoz-Robredo et al., 2013), as well as promotes the expression of *VvETR2* and *VvCTR1* (Chervin and Deluc, 2010). On the contrary, the ethylene inhibitor 1-MCP can significantly suppress ethylene biosynthesis and the rise in respiratory rate, reduce anthocyanin accumulation, inhibit fruit expansion and the reduction of acidity (Chervin et al., 2004), and suppress the expression of sugar transporters (e.g., *SUC11* and *SUC12*) and ethanol dehydrogenases (Tesniere et al., 2004; Chervin et al., 2006). 1-MCP treatment also markedly affects grape storage quality by decreasing respiration and ethylene production, reducing rachis browning and chlorophyll degradation, and maintaining higher anthocyanin content and lower ester content (Silva et al., 2013; Wang et al., 2019). These studies indicate that ethylene regulates fruit coloring, the formation of quality- and flavor-associated substances, and the visual and nutritional quality of grapes during storage.

Ethylene biosynthesis genes and key genes in the ethylene signaling pathway exhibit different expression levels during different grape developmental stages. The highest ACC oxidase transcript abundance is observed immediately before véraison, which suggests that peak ethylene production occurs before véraison (Deluc et al., 2007; Pilati et al., 2007). *VviERF045* expression gradually increases before véraison and peaks during the ripening stage (Leida et al., 2016). The transcript abundance of *ERF6* transcription factors is significantly affected by ripening and correlated with the transcript abundance of terpene synthases and lipoxygenases involved in flavor formation (Cramer et al., 2014). In addition, both *VvACO4* and *VvEIL3* regulate ethylene synthesis and fruit ripening, and over-expression of *VvACO4* and *VvEIL3* shows a significant ethylene production and accelerates fruit ripening compared to control fruits (Wang et al., 2022). These findings suggest that the ethylene signaling pathway plays an important role in the ripening process of grape berries.

### 4.3 Ethylene regulates the ripening and softening of oranges

Citrus is also a non-climacteric fruit and lacks an ethylene-induced respiratory peak and climacteric rise in ethylene production (Chen et al., 2018). However, a previous study showed that citrus fruits exhibit “pseudoclimacteric” behavior and that young citrus fruitlets attached to the tree produce high levels of ethylene, which decrease dramatically toward the end of stage I and thereafter (**Figure 3D**). Moreover, citrus exhibits high sensitivity to ethylene at stage I, and exogenous ethylene treatment advances and increases ethylene production as in climacteric fruits (Katz et al., 2004). Previous studies suggest that ethylene biosynthesis can be divided into two types: system I and system II. System I ethylene biosynthesis mainly occurs before the maturation of climacteric fruits and throughout the ripening stage of non-climacteric fruits, with a small amount of ethylene synthesized. In contrast, system II ethylene biosynthesis mainly takes place during the ripening stage of climacteric fruits. Orange development may involve two ethylene biosynthesis pathways: system I-like and system II-like (Katz et al., 2004). *CsACS2* plays a significant role in system I-like ethylene production, while *CsACS1* is involved in system II-like ethylene production (Katz et al., 2004; Kashyap and Banu, 2019).

To improve skin color in early season or early harvest cultivars, ethylene degreening treatment is widely used to promote chlorophyll degradation and carotenoid accumulation (Zhou et al., 2010; Fauziah and Bintoro, 2021). The increased accumulation of carotenoid pigments in citrus fruits observed following ethylene or ethephon treatment is due to the induction of carotenoid biosynthesis genes (Rodrigo and Zacarias, 2007; Matsumoto et al., 2009; Huang et al., 2021). In addition, the repression of β-carotene hydroxylase genes is significantly increased by ethylene or ethephon, thereby leading to the preferential accumulation of β-carotene and β-cryptoxanthin (both of which contribute to orange coloration) (Zhou et al., 2010). Exposure to ethylene can also stimulate various adaptation and metabolic processes, which can impact fruit internal and nutritional quality (Mayuoni et al., 2011), thereby regulating citrus fruit ripening (Katz et al., 2004; Ding et al., 2015; Li et al., 2019). Moreover, ethylene can promote the accumulation of aroma substances in orange fruit (Sharon-Asa et al., 2003), while 1-MCP delays coloring and inhibits ripening (Cai et al., 2006). These findings indicate that citrus fruit ripening is mediated by ethylene.

### 4.4 Ethylene regulates the ripening and softening of other non-climacteric fruits

Loquat, litchi, sweet cherry, longan, ananas, blueberry, and watermelon are all defined as non-climacteric fruits. Ethylene production increases slightly during the coloring stage in loquat fruits, and ethylene is also essential for peel coloration and carotenoid biosynthesis (Alós et al., 2019). Treating loquats with exogenous ethylene during the véraison or post-harvest stage significantly promotes the expression of the *ACO1* gene and the biosynthesis of endogenous ethylene (Alós et al., 2017). Conversely, 1-MCP treatment inhibits the expression of ethylene biosynthesis genes, delays the ripening process, and reduces the activities of lipoxidase (LOX) and peroxidase (POD) in post-harvest loquat fruits (Liguori et al., 2015; Alós et al., 2017). Additionally, 1-MCP suppresses the accumulation of reactive oxygen species and slows the oxidation of phenols, thus inhibiting fruit rot (Cai et al., 2006). In peppers and jujubes, recent studies have shown that ethylene plays an important role in ripening and post-harvest storage. In these species, the expressions of certain *ACS* genes, including *CaACS1*, *CaACS2*, *ZjACS2*, *ZjACS3*, *ZjACS5*, and *ZjACS7*, were altered alongside ethylene production during fruit ripening (Aizat et al., 2013; Zhang et al., 2018). In sweet cherries, ethylene biosynthesis increases significantly during ripening, and ethylene can increase the respiration rate (Gong et al., 2002). Meanwhile, 1-MCP treatment inhibits the rotting of post-harvest cherries (Mozetič et al., 2006) by decreasing the respiratory rate and enhancing the activity of superoxide dismutase (SOD) (Sharma et al., 2010). 1-MCP also delays the reduction of POD and catalase (CAT) activities and reduces the malondialdehyde (MDA) content (Yang et al., 2011). Additionally, high-concentration exogenous ethylene treatment can facilitate chlorophyll degradation and anthocyanin biosynthesis in litchi fruits (Wang et al., 2007), while 1-MCP treatment suppresses the browning of post-harvest litchi and ananas fruits, maintaining their quality (Selvarajah et al., 2001; Sivakumar and Korsten, 2010). Although raspberries are non-climacteric fruits, increased ethylene production and respiration rate were detected at the white-fruit stage and continued to increase until maturity. Treatment of raspberries with 1-MCP at the white stage delayed loss of firmness, suggesting that softening may be partially regulated by ethylene in raspberries (Fuentes et al., 2015). Blueberries are also known as non-climacteric fruits, although ethylene is reported to be involved in their ripening process (Watanabe et al., 2021). Ethylene absorbent treatment can reduce weight loss and decay, as well as prevent the loss of total phenolic content and maintain firmness, in blueberries (Wang et al., 2018). Furthermore, pre-treatment with 1-MCP prevents berry softening and cell wall degradation in blueberries (Ortiz et al., 2018). Longan fruits are also non-climacteric and exhibit minimal changes in soluble solids content (SSC) and titratable acidity (TA) after harvest (Jiang et al., 2002). However, there has been an increase in ethylene production associated with post-harvest decay for longan fruits stored at 20°C (Wall et al., 2011), and the ethylene-responsive factor-like gene DlERF1 has been reported to regulate senescence-associated gene expression in longan fruits (Kuang et al., 2012). In watermelons, the expressions of two *ACS* isoforms and two *ACO* isoforms were significantly upregulated during ripening, indicating that ethylene biosynthesis genes could potentially play important roles in the ripening process of watermelon flesh (Zhou et al., 2016). Watermelons are also sensitive to exogenous ethylene, which can promote maturation (Mao et al., 2004) and induce the appearance of the water-soaking phenomenon by inducing the expression of softening-related genes (Karakurt and Huber, 2004). Furthermore, exogenous ethylene treatment of fresh-cut watermelons can increase the respiration rate and decrease fruit quality (Saftner et al., 2007). These findings suggest that the development and ripening of non-climacteric fruits may be at least partially regulated by ethylene.

## 5 Ethylene interacts with other hormones to regulate the ripening and softening of non-climacteric fruits

**Figure 4:**
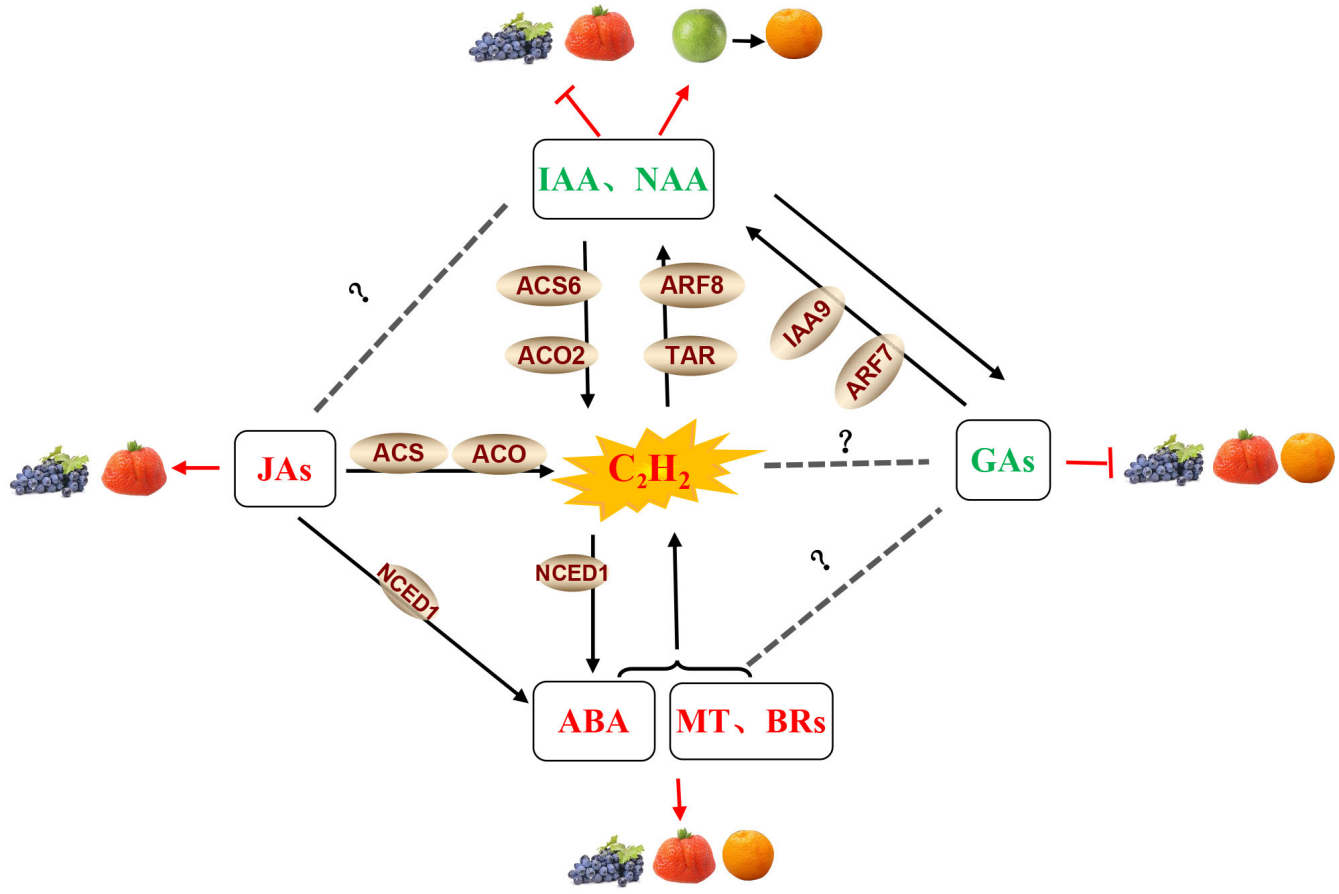
Phytohormone crosstalk between ethylene and other hormones in grape, strawberry, and citrus fruits. Note: The hormones and their related components involved in fruit ripening shown in the figure are abscisic acid (ABA), auxin (IAA),1-naphthaleneacetic acid (NAA), ethylene (C_2_H_4_), brassinosteroids (BRs), gibberellins (GAs), melatonin (MT) and jasmonates (JAs), tryptophan aminotransferase (TAR), auxin response factor (ARF), an auxin/indole-3-acetic acid (Aux/IAA) protein (IAA9), 9-*cis*-epoxycarotenoid dioxygenase 1 (NCED1), 1-aminocyclopropane-1-carboxylic acid synthase (ACS), and 1-aminocyclopropane-1-carboxylic acid oxidase (ACO). JA, ABA, BRs, and MT in the red fond have a positive effect on fruit ripening (Davies et al., 2006; Sun et al., 2010; Liu et al., 2011; Concha et al., 2013; Delgado et al., 2018; Garrido-Bigotes et al., 2018; Mansouri et al., 2021; Xia et al., 2021); Gas and IAA/NAA in the green fond had a negative effect on fruit ripening (Böttcher et al., 2011; Liu et al., 2011; Ma et al., 2021a, b; Tyagi et al., 2022); and jasmonate-activated fruit ripening is possibly associated with the stimulation of ethylene biosynthesis by an increase in ACO and ACS activities (Mukkun and Singh, 2009), while ABA, BRs, and MT can also stimulate ethylene production (Jiang and Joyce, 2003; Xu et al., 2018). C_2_H_4_ and IAA/NAA have a mutually reinforcing relationship. NAA can strongly upregulate *ACS6* and *ACO2* to improve ethylene biosynthesis (Ziliotto et al., 2012). In turn, the elevated concentrations of ethylene may lead to the induction of TAR expression, thus increasing the production of IAA (Böttcher et al., 2013). Ethylene also induces the transcription of *VvNCED1* and the synthesis of ABA (Sun et al., 2010). The crosstalk between JAs and ABA, as well as GAs and IAA/NAA, also exists in non-climacteric fruit (Jung et al., 2014; Wang et al., 2015).

Several other phytohormones may also contribute to regulating the ripening process in non-climacteric fruits, as shown in **Figure 4**. Recent reports have revealed that abscisic acid (ABA) participates in the regulation of non-climacteric fruit ripening, including in grapes (Jia et al., 2017), strawberries (Jia et al., 2011), citrus (Rodrigo et al., 2006; Wang et al., 2016), and watermelons (Wang et al., 2017). There are many examples of hormone crosstalk in plant growth regulation. For example, ethylene and ABA interact to induce flowering in *Pharbitis nil* (Arc et al., 2013), mediate the effects of soil compaction on shoot growth (Hussain et al., 2000), induce hyponastic growth in *A. thaliana* (Benschop et al., 2007), and regulate climacteric fruit ripening (Qiao et al., 2021). These studies have shown that the relationship between ABA and ethylene is antagonistic. Generally, the ripening of climacteric fruits is controlled by ethylene, while non-climacteric fruit ripening is mainly regulated by ABA (Bai et al., 2021). However, several studies have reported that ethylene also participates in non-climacteric fruit ripening by interacting with ABA. In post-harvest strawberries, ethylene facilitates ABA accumulation in receptacle tissue (Tosetti et al., 2020). In grapes, functional interaction and synergism between ABA and ethylene at the onset of ripening have been observed. Endogenous ethylene induces the transcription of *VvNCED1* and the synthesis of ABA, and both ethylene and ABA are likely to be important and required to initiate the process of berry ripening (Sun et al., 2010). ABA treatment can stimulate ethylene production in strawberries (Jiang and Joyce, 2003). Thus, the interaction of ABA with ethylene appears to play a vital role in non-climacteric fruit ripening. Similar results from the effects of jasmonates (JAs) in the non-climacteric fruit ripening process have also been reported. JAs have been found to be involved in fruit ripening and to upregulate the phenylpropanoid pathway in strawberries (Concha et al., 2013; Delgado et al., 2018; Garrido-Bigotes et al., 2018) and grapes (Liu et al., 2011). Moreover, JA-activated fruit ripening is possibly associated with the stimulation of ethylene biosynthesis via an increase in ACO and ACS activities (Mukkun and Singh, 2009). These results suggest that JA is involved in strawberry fruit ripening in an ethylene-dependent manner.

It is well known that ripening can be delayed by the application of auxins to grapes before véraison (Böttcher et al., 2011), as well as strawberries (Liu et al., 2011). Auxin and 1-naphthaleneacetic acid (NAA) can induce carotenoid accumulation in citrus fruits (Ma et al., 2021a, b). Elevated concentrations of ethylene prior to the initiation of ripening may induce the expression of the auxin biosynthesis-related gene *TAR*, thus increasing the production of indole-3-acetic acid (IAA) during grape berry ripening (Böttcher et al., 2013). In addition, pre-véraison NAA treatment strongly upregulated the expression of the *ACS6* and *ACO2* genes (Ziliotto et al., 2012), indicating the existence of crosstalk between ethylene and auxin in non-climacteric fruit (**Figure 4**). Meanwhile, an antagonistic relationship between auxin and gibberellic acid (GA) has also been reported. Treatment with GA at the pre-bloom stage decreases the expression of *VvIAA9* and *VvARF7* and partially activates auxin signaling in grapes (Jung et al., 2014). Exogenous application of the auxin analog 4-chlorophenoxyacetic acid (4-CPA) to grapes promoted the biosynthesis of GA_3_ (Lu et al., 2016). These results suggest interactions between GAs and auxin signaling in non-climacteric fruit (**Figure 4**). Additionally, GA treatment of grapes delayed sugar accumulation, acid degradation, and color development (Tyagi et al., 2022) and also delayed the color break and harvest date (Alós et al., 2006; Fujii et al., 2008). As such, the effect of GA on delayed ripening is possibly associated with the activation of auxin signaling. However, our knowledge about the interactions of gibberellins with ethylene and ABA in the ripening process needs to be deciphered. Moreover, previous studies reported that the application of brassinosteroids (BRs) and melatonin to grapes and strawberries significantly promoted fruit ripening (Davies et al., 2006; Mansouri et al., 2021; Xia et al., 2021). An antagonistic interaction between melatonin and ethylene has been reported, and melatonin application coordinates with ethylene biosynthesis to regulate grape ripening (Xu et al., 2018). Similarly, the application of brassinolides (BRs) is known to induce fruit ripening in some climacteric fruits (Zhu et al., 2015; Liu et al., 2022), and as such, it will be important to investigate the relationship between the BRs and ethylene biosynthesis in non-climacteric fruits. Meantime, further research should be conducted to clarify such multi-hormonal regulatory mechanisms in relation to the ripening and softening of non-climacteric fruits.

## 6 Conclusions and perspectives

There have been considerable advances in our understanding of the important role of ethylene in regulating the ripening and softening of non-climacteric fruits. It appears that endogenous and exogenous ethylene mediates changes in numerous ripening- and softening-related qualities, such as color, texture, aroma, and flavor. An array of genes associated with the ethylene signaling pathway have been found to regulate the ripening and softening of non-climacteric fruits. In addition, the antagonistic interaction between multiple hormones appears to be associated with the ripening and softening of non-climacteric fruits. These findings clearly show that ethylene biosynthesis and signal transduction are regulated in a variety of ways, many of which are linked with the processes of ripening and softening, in non-climacteric fruits.

Because many of the regulators of fruit ripening and softening are shared among both climacteric and non-climacteric fruits, the comprehensive and detailed molecular regulatory mechanism for the regulation of ethylene signaling in the ripening and softening of non-climacteric fruit remains to be further explored. We suggest the following as important research priorities and questions requiring answers:

- Certain transcription factors play a role upstream of the ethylene biosynthesis pathway and participate in ethylene biosynthesis. However, little is known about the regulatory relationships between these transcription factors, including MADS-RIN, NAC, and ERFs, and the *ACS* and *ACO* genes in non-climacteric fruits.
- The structures and protein phosphorylation mechanisms of ethylene receptor genes involved in the ripening and softening of non-climacteric fruits require validation.
- With the exception of certain ethylene biosynthesis genes and ethylene receptors, the expression patterns and functions of other ripening- and softening-associated transcription factors, such as RAN1, EIN2, EIN3, and EBF, should be characterized in non-climacteric fruits.
- Ethylene exhibits complex interactions with other phytohormones, including ABA and IAA. Research should be conducted to better understand how these phytohormones jointly regulate the ripening process in non-climacteric fruits.
- Abiotic stressors, such as drought, cold injury, high temperature, and other environmental factors, play an important role in regulating the expression of *ACS* and *ACO*. However, the mechanisms underlying how these abiotic stressors regulate the ethylene biosynthesis in non-climacteric fruits remain to be explored.
- Establishing the complete transcriptional regulatory network underlying the ripening and softening of non-climacteric fruits, as well as exploring key regulatory components, will promote the development of preservation technologies for non-climacteric fruits using molecular biological approaches.

## Author contributions

ML: Formal analysis, Writing – original draft, Data curation, Methodology. CW: Writing – review & editing. MS: Formal analysis, Methodology, Writing – original draft. HJ: Formal analysis, Methodology, Writing – original draft. TL: Data curation, Formal analysis, Writing – original draft. JW: Writing – review & editing. HC: Conceptualization, Supervision, Validation, Writing – review & editing, Formal analysis. QZ: Conceptualization, Funding acquisition, Supervision, Writing – review & editing.
